# Complete Genome Sequence of Mycoplasma feriruminatoris Strain IVB14/OD_0535, Isolated from an Alpine Ibex in a Swiss Zoo

**DOI:** 10.1128/MRA.01528-19

**Published:** 2020-03-19

**Authors:** Fabien Labroussaa, Andreas Thomann, Pamela Nicholson, Laurent Falquet, Joerg Jores

**Affiliations:** a Institute of Veterinary Bacteriology, University of Bern, Bern, Switzerland; b Biochemistry Unit, University of Fribourg and Swiss Institute of Bioinformatics, Fribourg, Switzerland; Portland State University

## Abstract

Mycoplasma feriruminatoris is a fast-growing and genetically tractable mycoplasma species. We sequenced the Swiss strain IVB14/OD_0535, isolated from an Alpine ibex. This strain has a circular genome of 1,027,435 bp with a G+C content of 24.3%. It encodes 835 open reading frames (ORFs), 2 rRNA operons, and 30 tRNAs.

## ANNOUNCEMENT

Mycoplasma feriruminatoris is phylogenetically closely related to the Mycoplasma mycoides cluster (1), which encompasses the etiological agents of contagious bovine (CBPP) and caprine pleuropneumonia (CCPP), two of the most severe diseases affecting cattle and goats, respectively. To date, the mode of infection and pathogenicity of *M. feriruminatoris* toward wild and domestic ruminants are still unclear.

*M. feriruminatoris* strain IVB14/OD_0535 was isolated in 2014 from the liver, spleen, and kidney of a 2-month-old Alpine ibex (Capra ibex) which died of septicemia at the Tierpark Dählhölzli (Bern, Switzerland). The organs were cultured at 37°C on tryptone soy agar (TSA-SB; Becton, Dickinson) supplemented with 5% (vol/vol) sheep blood. Mycoplasma-like colonies were subcultivated on mycoplasma agar (MS; Mycoplasma Experience Ltd.), filter cloned three consecutive times, and identified as Mycoplasma feriruminatoris using matrix-assisted laser desorption ionization–time of flight (MALDI-TOF) mass spectrometry (Microflex LT/SH system; Bruker).

Genomic DNA was extracted using phenol-chloroform extraction and isopropanol precipitation (2). The quality and quantity of the genomic DNA were assessed on agarose gel and using the Qubit fluorometer (Invitrogen). High-molecular-weight DNA was sheared in a Covaris g-TUBE (Covaris) to obtain 10-kb fragments. DNA was used to prepare a barcoded SMRTbell library with the PacBio SMRTbell template prep kit v.1 (Pacific Biosciences) or an Illumina library with the Nextera DNA Flex library prep kit (Illumina, Inc.) according to the manufacturer’s recommendations. Sequencing was done at the Lausanne Genomic Technologies Facility using both the PacBio Sequel and MiSeq systems. The genome was assembled from the PacBio reads (101,057 reads; average length, 6,554 bp; 662× coverage) in one unique contig using Canu v.1.8 (3), circularized with Minimus2 v.3.1 (4), and polished for 3 rounds with the Arrow software v.6.0.0.47841. One final round of polishing was performed with Pilon v.1.22 (5) using the Illumina reads (18,758,564 reads; 251 bp, paired end; raw coverage of 9,165×) in order to correct any possible base-calling errors. The genome was rotated to the first nucleotide of the start codon of the *dnaA* gene. The genome sequence was then annotated using Prokka v.1.13 (6).

The genome consists of a 1,027,435-bp chromosome with a G+C content of 24.3%. It encodes 835 open reading frames (ORFs), 2 rRNA operons, and 30 tRNAs. The genetic content of the IVB14/OD_0535 strain is very similar to the genome sequence of *M. feriruminatoris* G5847^T^ (7), whose genome was sequenced and published as a draft (88 contigs) in 2013 (8). Of the 835 coding sequences, 680 have assigned functions based on similarities with related mycoplasma proteins in UniProtKB. The remaining 155 are hypothetical proteins.

Given its unique growth attributes and the emergence of several groundbreaking synthetic genomics techniques (9–11) allowing the precise engineering of mycoplasma genomes on a large scale (12), *M. feriruminatoris* has the potential to be a workhorse for many research and industrial applications.

### Data availability.

The annotated genome sequence was deposited in DDBJ/ENA/GenBank under the accession number LR739236 and project number PRJEB35485. The raw reads generated from the PacBio and Illumina sequencing were deposited under the accession numbers ERR3938361 and ERR3938362, respectively.
